# From Africa to Europe and back: refugia and range shifts cause high genetic differentiation in the Marbled White butterfly *Melanargia galathea*

**DOI:** 10.1186/1471-2148-11-215

**Published:** 2011-07-21

**Authors:** Jan C Habel, Luc Lens, Dennis Rödder, Thomas Schmitt

**Affiliations:** 1 Musée national d'histoire naturelle Luxembourg, L-2160 Luxembourg, Luxembourg; 2 Terrestrial Ecology Unit, Department of Biology, Gent University, B-9000 Gent, Belgium; 3 Department of Biogeography, Trier University, D-54296 Trier, Germany; 4 Zoologisches Forschungsmuseum Alexander Koenig, Adenauerallee 160, 53113 Bonn, Germany

**Keywords:** climatic oscillations, barriers, phylogeography, *Melanargia galathea*, *Melanargia lachesis*, allozyme electrophoresis, climate envelope modelling

## Abstract

**Background:**

The glacial-interglacial oscillations caused severe range modifications of biota. Thermophilic species became extinct in the North and survived in southern retreats, e.g. the Mediterranean Basin. These repeated extinction and (re)colonisation events led to long-term isolation and intermixing of populations and thus resulted in strong genetic imprints in many European species therefore being composed of several genetic lineages. To better understand these cycles of repeated expansion and retraction, we selected the Marbled White butterfly *Melanargia galathea*. Fourty-one populations scattered over Europe and the Maghreb and one population of the sibling taxon *M. lachesis *were analysed using allozyme electrophoresis.

**Results:**

We obtained seven distinct lineages applying neighbour joining and STRUCTURE analyses: (i) Morocco, (ii) Tunisia, (iii) Sicily, (iv) Italy and southern France, (v) eastern Balkans extending to Central Europe, (vi) western Balkans with western Carpathian Basin as well as (vii) south-western Alps. The hierarchy of these splits is well matching the chronology of glacial and interglacial cycles since the Günz ice age starting with an initial split between the *galathea *group in North Africa and the *lachesis *group in Iberia. These genetic structures were compared with past distribution patterns during the last glacial stage calculated with distribution models.

**Conclusions:**

Both methods suggest climatically suitable areas in the Maghreb and the southern European peninsulas with distinct refugia during the last glacial period and underpin strong range expansions to the North during the Postglacial. However, the allozyme patterns reveal biogeographical structures not detected by distribution modelling as two distinct refugia in the Maghreb, two or more distinct refugia at the Balkans and a close link between the eastern Maghreb and Sicily. Furthermore, the genetically highly diverse western Maghreb might have acted as source or speciation centre of this taxon, while the eastern, genetically impoverished Maghreb population might result from a relatively recent recolonisation from Europe via Sicily.

## Background

The impacts of climatic oscillations on the earth's biota have been intensively studied [1]. In the western Palaearctic, thermophilic organisms went extinct over major parts of Central and North Europe during cold stages and survived in the lowlands of lower latitudes in often distinct refugia [2-6]. Molecular studies revealed that most of these taxa exclusively survived glacial periods south of the European high mountain chains in the Iberian, Italian and Balkan peninsulas, and some even in additional extra-Mediterranean refugia [7,8]. The long-term isolation of populations in these retreats over many thousands of years resulted in genetic differentiation [5]. During the warmer interglacial periods, species expanded their distribution ranges northwards and extended their different genetic lineages over more northern areas [9,10].

In contrast to the three more intensively studied Mediterranean refugia of southern Europe (Iberia, peninsular Italy and the Balkans), little is known about North African refugia and the biogeographical relation between the Maghreb and southern Europe separated by the two narrow sea straits of Gibraltar and Sicily. It has been shown that the Maghreb is often sub-structured following an east-west [e.g. [11-13]] or south-north differentiation pattern [e.g. [5,14]]; in some cases, genetic continuity was demonstrated between the Maghreb and Sicily [e.g. [15,16]]. Other studies underline the important role of Sicily as diversification centre for European taxa unravelling deep genetic splits between this island and peninsular Italy (e.g. *Erinaceus europaeus*: [17]; *Pseudepidalea viridis*: [18,19]). Few molecular analyses also reveal the outstanding importance of North Africa as a refugium for thermophilic species during glacial periods [e.g. [12,14,20,21]]. However, most studies focus either on the Maghreb or the southern European refugia and do not combine the distribution of species all over north-western Africa and throughout Europe.

To study the biogeographical importance of the Maghreb region and its connection with Europe, we selected the Marbled White butterfly species complex *Melanargia galathea *(Linnaeus, 1758) and *Melanargia lachesis *(Hübner, 1790) as a model system using two analytical tools (allozyme polymorphisms and distribution modelling). Today, *M. galathea *is widely distributed from the Maghreb region (mountain ranges of Morocco, Algeria and Tunisia) [22,23] to the English Midlands [24], and from the Pyrenees [25] to the Baltic Sea in Poland [26]. On the Iberian Peninsula, *M. galathea *is replaced by its sibling species *M. lachesis*. Thus, the Italian peninsula is the only possible link between North Africa and Europe for *M. galathea*.

Previous molecular studies on these butterflies based on allozyme polymorphisms supported the sibling species status of both taxa and revealed two genetic groups in *M. galathea*, one western and one eastern group, indicating an Italian and a Balkan refugium [27], with further substructures in the Balkan region [28]. Preliminary data for the western Maghreb showed the highest known values of genetic variability in this region [29]. Based on these data, we hypothesized a Maghreb origin of the species and colonisation of Europe via Sicily and Italy. However, these previous studies lack populations from the eastern Maghreb, Sicily, Italy and southern France. In this article we combine an allozyme data set covering most of the recent distribution of the species with climate envelope models to test the previously postulated biogeographical scenario of refugia and barriers during the last ice ages until today, addressing the following questions:

(i) Which refugia are of importance for the glacial survival of the *M. galathea */*lachesis *species complex during the subsequent glacial periods?

(ii) Is there any evidence of genetic structuring within the North African and Italian refugia?

(iii) Which routes of expansion and retraction followed the butterfly throughout time?

## Results

### Phylogeographic analyses

All enzyme loci had banding patterns consistent with known quaternary structures. While most loci were inherited autosomally, 6PGDH and ME were located on the Z chromosome so that hemizygous females (but not males) had a single copy [27]. No general linkage disequilibrium was observed for any locus (all *p *> 0.05 after Bonferoni correction). A total of 13 analysed loci were polymorphic, but two loci (FUM, GPDH) were monomorphic throughout all samples. Allele frequencies for each enzyme and population are given in an additional file 1.

When calculating parameters of genetic diversity, all 15 loci were used. The genetic diversities of populations showed strong variability among populations of different regions, and standard deviations were high if compared against means (ratio standard deviation against means: *A *9.0%; *H*_e _12.9%; *H*_o _18.3%; *P*_tot _18.1%; *P*_95 _15.6%). Values for all populations analysed are given in Table 1, overall means in Table 2.

**Table 1:** Sampling location and five parameters of genetic diversity for 41 populations of *Melanargia galathe a *from its western Palaearctic distribution area and one population of *M. lachesis *from the Pyrenees: number of individuals analysed (*N*), mean number of alleles per locus (*A*), percentage of expected (*H_e_*) and observed (*H_o_*) heterozygosity, percentage of polymorphic loci not exceeding 95% (*P*_95_) and total number of polymorphic loci (*P*_tot_)

Region	Location	**Running Nr**.	Long. (N) Lat. (E/W)	Date of sampling	*N*	*A*	*H_e _*(%)	*H_o _*(%)	*P*_95 _(%)	*P*_tot _(%)
**Morocco**	M-Oukaimeden	1	31.12; 7.52W	23-V-05	36	2.33	20.5	20.6	46.7	46.7
	M-Naour	2	32.29; 5.56W	26-V-05	36	2.33	17.0	13.5	33.3	53.3
	M-Bekrite	3	33.05; 5.13W	29-V-05	36	2.53	17.2	18.5	33.3	53.3
	M-Timhadite	4	33.14; 5.03W	29-V-05	36	2.33	18.7	17.7	33.3	60.0

**Tunisia**	T-Ain Draham	5	36.46; 8.42E	23-V-10	27	2.20	17.6	15.6	40.0	53.3
	T-Nebeur	6	36.18; 8.46E	26-V-10	40	2.00	12.3	9.9	26.7	60.0
	T-Table de Yagurta	7	35.45; 8.23E	25-V-10	40	2.13	13.6	10.6	40.0	53.3
	T-Thala	8	35.34; 8.41E	25-V-10	40	1.80	12.5	12.1	26.7	40.0
	T-Béja	9	36.44; 8.54E	28-V-10	40	1.86	13.6	9.8	33.3	40.0

**Sicily**	I-Valledomo	10	37.45; 13.53E	4-VII-07	40	2.13	14.5	13.2	40.0	53.3
	I-Francavilla	11	37.53; 15.07E	5-VII-07	30	2.26	17.8	14.8	26.7	66.7
	I-Reitano	12	37.58; 14.20E	26-VI-07	40	2.00	15.0	12.2	33.3	46.7

**Italy**	I-St. Giorgio	13	38.17; 15.59E	6-VII-07	40	2.13	17.1	17.2	33.3	40.0
	I-Mormanno	14	39.54; 15.58E	7-VII-07	40	2.66	21.3	15.6	53.3	73.3
	I-Napoli	15	41.08; 14.19E	7-VII-07	20	2.13	18.2	13.3	40.0	46.7
	I-Rieti	16	42.25; 12.53E	8-VII-07	40	2.20	17.8	17.4	33.3	53.3
	I-Consuma	17	43.48; 11.36E	8-VII-07	40	2.20	19.2	17.5	40.0	60.0
	I-Verona	18	45.31; 10.55E	9-VII-07	40	2.13	17.7	13.5	40.0	46.7

**France**	F-Col de Tende	19	44.09;7.34E	31-VII-03	40	2.13	17.7	12.1	33.3	40.0
	F-Condat	20	45.20; 2.45E	11-VII-08	27	1.86	13.8	10.6	26.7	46.7
	F-Lorry	21	49.00; 6.06E	17-VI-03	40	2.06	17.6	15.3	33.3	40.0

**Central Europe**	L-Niederanven	22	49.39; 6.16E	13-VI-03	32	2.00	18.4	17.1	33.3	40.0
	D-Niederehe	23	50.18; 6.48E	18-VII-03	18	1.86	18.2	16.7	33.3	33.3
	D-Bossler	24	48.36; 9.36E	VII-04	40	2.00	18.5	17.0	40.0	40.0
	D-Buchenberg	25	50.43; 11.40E	VII-03	40	2.33	19.3	16.4	33.3	40.0
	A-Jadersdorf	26	46.40; 13.18E	18-VIII-04	40	2.00	13.5	12.6	33.3	33.3
	A-Hochobir	27	46.30; 14.30E	23-VIII-04	40	2.06	14.6	13.3	26.7	46.7
	A-Schöckl	28	47.12; 15.28E	22-VIII-04	40	2.13	17.0	15.7	33.3	40.0
	A-Leithagebirge	29	47.57; 16.39E	31-VII-03	14	1.86	14.8	13.6	33.3	40.0

**Hungary**	H-Csákvár	30	47.24; 18.26E	13-VII-04	40	2.33	17.2	15.9	33.3	46.7

**western Balkans**	SLO-Postojna	31	45.47; 14.12E	15-VII-04	40	2.00	14.4	13.5	33.3	40.0
	MNE-Durmitor	32	43.10; 19.08E	10-VII-03	12	1.86	14.8	14.5	26.7	33.3

**Romania**	RO-Hoteni	33	47.38; 24.02E	25-VII-04	40	1.93	17.9	20.5	33.3	33.3
	RO-Cluj	34	46.45; 23.35E	23-VII-04	40	2.13	18.3	17.7	40.0	40.0
	RO-Voslobeni	35	46.40; 25.38E	29-VII-04	40	1.86	18.5	18.6	33.3	40.0
	RO-Pasul Predelus	36	45.35; 26.09E	31-VII-04	40	2.33	20.4	18.8	33.3	40.0
	RO-Porta di Fier Transilvanici	37	45.31; 22.39E	15-VII-04	40	2.06	19.1	19.2	33.3	40.0
	RO-Inelet	38	44.58; 22.29E	9-VIII-04	19	1.80	19.2	16.0	33.3	40.0

**Bulgaria**	BG-Milanovo	39	43.05; 23.23E	10-VIII-04	40	2.06	17.8	17.3	33.3	40.0
	BG-Karandila	40	42.43; 26.20E	3-VIII-04	40	2.06	18.4	17.1	33.3	40.0
	BG-Trigrad	41	41.36; 24.17E	7-VIII-04	40	2.06	16.5	16.5	33.3	33.3

***lachesis***	E-Col de Perbes*	42	42.23; 1.13E	19-VII-03	40	2.26	20.8	17.6	40.0	40.0

* *M. lachesis*

**Table 2:** Means of sample sizes and genetic diversities of the different genetic groups of *Melanargia galathea *and *M. lachesis*; *p *values of Kruskal Wallis ANOVAs among groups are given

	*N*	*A*	*H*_e_	*H*_o_	*P*_95_	*P*_tot_
All	36.0 ± 7.9	2.10 ± 0.19	17.0 ± 2.2	15.3 ± 2.8	34.5 ± 5.4	45.2 ± 9.4

Morocco	36.0 ± 0.0	2.38 ± 0.10	18.4 ± 1.6	17.6 ± 3.0	36.7 ± 6.7	53.3 ± 5.4
Tunisia	37.4 ± 7.8	2.00 ± 0.17	13.9 ± 2.1	11.6 ± 2.4	33.3 ± 6.7	49.3 ± 8.9
Sicily	37.7 ± 5.8	2.13 ± 0.13	15.8 ± 1.8	13.4 ± 1.3	33.3 ± 6.6	55.6 ± 10.2
Italy + SE France	35.3 ± 8.3	2.19 ± 0.24	17.9 ± 2.3	15.1 ± 2.6	38.1 ± 8.3	52.4 ± 11.2
SW Alps	40	2.13	17.7	12.1	33.3	40.0
eastern Balkans + Central Europe	36.8 ± 7.4	2.04 ± 0.15	17.8 ± 1.7	16.9 ± 2.0	33.7 ± 2.9	38.7 ± 3.6
western Balkans	26.5 ± 15.6	2.01 ± 0.22	15.3 ± 1.3	14.4 ± 1.1	31.6 ± 3.3	40.0 ± 5.5
*p *(Kruskal Wallis ANOVA)	0.33	0.044	0.019	0.004	0.65	< 0.001

eastern Balkans + Romania	35.7 ± 8.4	2.05 ± 0.15	17.1 ± 2.2	15.5 ± 1.8	33.3 ± 3.8	39.0 ± 4.6
Central Europe	37.7 ± 7.0	2.03 ± 0.16	18.5 ± 1.1	18.0 ± 1.4	34.0 ± 2.2	38.5 ± 2.9
*p *(Kruskal Wallis ANOVA)	0.56	> 0.99	0.31	0.011	0.75	0.87

*M. lachesis*	40	2.26	20.8	17.6	40.0	40.0

**Figure 1:**
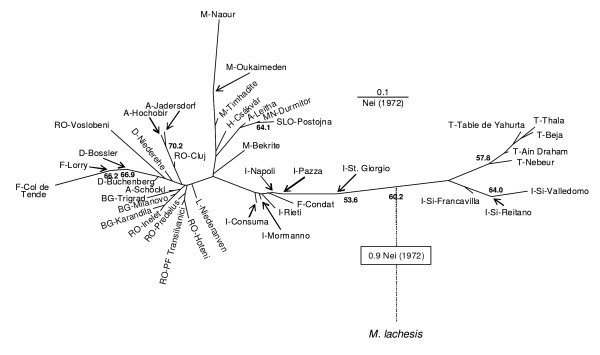
**Neighbour-joining dendrogram of *Melanargia galathea *and *M. lachesis***. The phenogram is based on the genetic distances (Nei, 1972) [56] of 41 populations of *M. galathea *and one population of *M. lachesis*.

A neighbour joining phenogram based on allele frequencies (Figure 1) showed a first split between *M. galathea *and *M. lachesis *with a genetic distance [28] of about 0.9. The second split between *M. galathea *populations from Tunisia and Sicily on the one hand and all remaining *M. galathea *populations on the other was in average about half of the genetic distance of the first split. The outgroup *M. lachesis *routing the tree also supports this split being the first one in *M. galathea*. The Tunisia - Sicily group showed a further genetic differentiation between these two geographic regions. All these splits are supported by bootstrap values. The remaining populations split into five groups, the populations from Morocco and four European groups: (i) mainland Italy and southern France (Condat), (ii) western Balkans and western Carpathian Basin, (iii) eastern Balkans, Romania and Central Europe, as well as (iv) south-western Alps (Col de Tende). The latter group is the only one well supported by bootstrapping. Unexpectedly, the populations from Morocco are not well distinguishable in this tree from the western Balkan group. Three populations are not matching any of these groups: the north-eastern French population Lorry and the southern German population Bossler are intermediate between groups iii and iv, and the southern Calabrian population St. Giorgio shows some traits of the Sicily-Tunisia group thus not clustering together with the other populations from mainland Italy. These three populations are thought to be of hybrid origin between the respective genetic groups.

**Figure 2:**
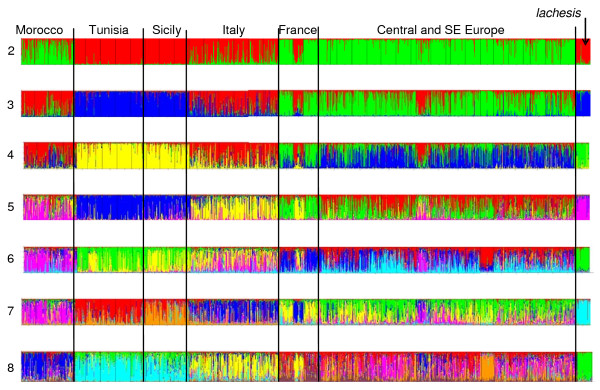
**Individual based genetic classification of *Melanargia galathea *and *M. lachesis***. Bayesian analysis of all *M. galathea *and *M. lachesis *populations performed using the STRUCTURE software [61]. Analyses for K = 2 to K = 8 are depicted.

STRUCTURE plots (for K = 2 to K = 8) support the topology of the neighbour joining phenogram (Figure 2); the sequence of splits of STRUCTURE groups is mostly reflecting the genetic distances in the tree. Additionally, the samples from Morocco are consistently separated as one group from all other *M. galathea *populations from K = 5 onwards. However, the STRUCTURE analysis is not able to distinguish the western Balkan group from the eastern Balkans, Romania and Central Europe group. Furthermore, STRUCTURE did not reflect the hybrid origin of three populations mentioned above: St. Giorgio (13) is grouped with all other populations from Italy, Bossler (24) with all populations from Central and south-eastern Europe and Lorry (21) is part of the south-western Alps group.

The overall differentiation among all populations including *M. lachesis *was strong (*F*_ST_: 0.179, *p *< 0.001); excluding this outgroup population only decreased this value marginally (*F*_ST_: 0.169, *p *< 0.001) (Table 3). Hierarchical variance analyses well supported the hierarchical structures of the neighbour joining phenogram and of STRUCTURE analyses (Table 4). Thus, these analyses strongly support (i) the genetic break in the Maghreb, (ii) the break between Sicily and mainland Italy, (iii) the differentiation into four genetic lineages in continental Europe, (iv) the cohesiveness between Sicily and Tunisia, (v) the lack of differentiation from the eastern Balkans via Romania to Central Europe and (vi) the strong genetic similarity between mainland Italy and southern France. The genetic differentiation within the seven groups at the lowest hierarchical level was low to moderate ranging from *F*_ST _values of 0.0142 to 0.0614 (Table 3).

**Table 3:** Analyses of molecular variance for all *Melanargia galathea *populations and one population of *M.lachesis*

Group	among populations (*F*_ST_)	among individuals within populations (*F*_IS_)	within individuals
All *galathea *and *lachesis*	0.1789*** (0.1889)	0.0875*** (0.0758)	(0.7910)
All *galathea*	0.1685*** (0.1767)	0.0858*** (0.0748)	(0.7968)
Sicily and Tunisia	0.0623*** (0.0527)	0.1111*** (0.0882)	(0.7059)
Sicily	0.0476*** (0.0345)	0.0827* (0.0570)	(0.6325)
Tunisia	0.0425*** (0.0381)	0.1252*** (0.1075)	(0.7513)
Morocco	0.0334*** (0.0304)	0.0408 (0.0358)	(0.8427)
Italy, Balkans, Central Europe	0.0951*** (0.0939)	0.0853*** (0.0763)	(0.8174)
Italy	0.0116 (0.0112)	0.1092*** (0.1034)	(0.8438)
Italy and S France	0.0142* (0.0135)	0.1143*** (0.1066)	(0.8258)
west Balkan group	0.0500*** (0.0411)	0.0999** (0.0780)	(0.7028)
east Balkan and Central Europe	0.0614*** (0.0580)	0.0673*** (0.0597)	(0.8282)

*F *statistics (top line) with their respective variance values (in parenthesis below). Groupings following neighbour joining analysis and STRUCTURE plots (see Figures 1 and 2).

*: *p *< 0.05; **: *p *< 0.01; ***: *p *< 0.001

**Table 4:** Hierarchical variance analyses of *Melanargia galathea *and *M.lachesis *among genetic groups

Groups	among groups (*F*_CT_)	within groups (*F*_SC_)	**prop. of among groups variance of total variance among pops**.
*galathea *vs *lachesis*	0.1811*** (0.2308)	0.1694*** (0.1768)	56.6%
Tunisia + Sicily vs rest of Europe	0.2256*** (0.2786)	0.0892*** (0.0852)	76.6%
Tunisia vs Morocco	0.2153*** (0.2475)	0.0387*** (0.0349)	87.6%
Tunisia vs Sicily	0.0338 (0.0291)	0.0445*** (0.0370)	44.0%
Sicily vs Italy	0.1797*** (0.1928)	0.0203*** (0.0179)	91.5%
Italy vs S France (Condat)	0.0107*** (0.0102)	0.0119*** (0.0113)	47.4%
Italy vs SW Alps (Col de Tende)	0.1726*** (0.1993)	0.0117*** (0.0112)	94.7%
east Balkans + Central Europe vs SW Alps (Col de Tende)	0.0615* (0.0621)	0.0611*** (0.0579)	51.8%
Italy vs SW Alps vs west Balkans vs east Balkans + Central Europe	0.0798*** (0.0813)	0.0479*** (0.0449)	64.4%
east Balkans + Romania vs Central Europe	0.0066 (0.0063)	0.0581*** (0.0548)	10.3%
Morocco vs continental Europe	0.0269* (0.0271)	0.0896*** (0.0877)	23.6%

*F *statistics (top line) with their respective variance values (in parenthesis below). Groupings following neighbour joining analysis and STRUCTURE plots (see Figures 1 and 2).

The genetic diversities among these genetic lineages showed significant differences (Table 2). Thus, the Morocco group showed the highest values achieved for *A, H*_e _as well as *H*_o_, and the means for *P*_95 _and *P*_tot _were above average. On the other extreme, Tunisia had the lowest means for *A, H*_e _and *H*_o_, and the mean for *P*_95 _was well below average; the genetic diversities of Tunisia were lower (*A, H*_e_, *H*_o_, *P*_tot_) or equal than in the otherwise rather similar populations from Sicily. The four groups from mainland Europe all have mostly intermediate genetic diversities scatted around the respective mean values.

### Species distribution modelling

According to the classification of Swets [30], we received 'excellent' AUC values in our 100 models (average training AUC = 0.927, average test AUC = 0.902). On average, the 'temperature annual range' had the highest explanatory power (30.3%), followed by the 'minimum temperature of the coldest month' (16.8%), the 'precipitation of the warmest quarter' (14.8%), the 'annual precipitation' (10.2%), the 'maximum temperature of the warmest month' (8.2%) and the 'precipitation of the driest quarter' (7.2%). All other variables contributed less than 5% each. The average minimum training presence was 0.05, and the lowest 10 percentile training omission threshold was 0.36.

**Figure 3:**
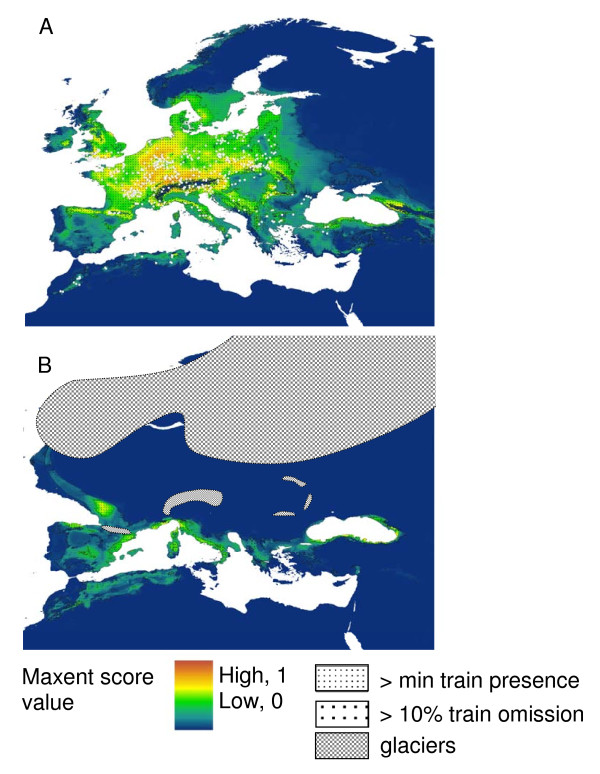
**Distribution modelling of *Melanargia galathea***. Potential distributions of *Melanargia galathea *under (a) current and (b) palaeoclimatic conditions (CCSM). Species records used for model training are indicated as white dots (3a). Dark grey areas refer to occurrence probabilities above the average minimum training presence; black areas represent those above the 10 percentile training omission.

The current potential distribution suggested by the SDM is highly coincident with the butterfly's recent range. The recent climatic niche over North Africa is displayed as two separate areas, in the West and East. Under palaeoclimatic conditions assumed to have prevailed 21,000 y BP (CCSM scenario), the potential distribution may have been much more restricted in Europe: Major parts of Central Europe changed into climatically unsuitable areas for *M. galathea *during the glacial period, while the southern European peninsulas (Iberia, Italy and the Balkan) retained suitable climatic conditions. Major parts of the Maghreb had a suitable climate for the butterfly being geographically more extended than today (Figure 3).

## Discussion

The obtained allozyme data displayed in neighbour-joining phenograms, structure plots and hierarchical variance analyses indicate a profound genetic split between the two taxa, *M. galathea *and *M. lachesis*. Nazari et al. [31] supported this pattern by three lines of evidence: (i) differences of the male genitalia between *M. lachesis *and *M. galathea*, (ii) a stronger difference in wing patterns between these two taxa than between *M. galathea *population in Europe and the Maghreb and (iii) remarkable differences in DNA sequences of the nuclear *wg *gene between *M. lachesis *and *M. galathea*, but no major differentiation between *M. galathea *samples from Europe and the Maghreb. However, the sequences of the two mtDNA genes *cox1 *and *16S *contradict the common pattern of allozymes, genital structures, wing patterns and nuclear DNA sequences: This marker is not well distinguishing *M. galathea *from Europe and *M. lachesis*, but shows remarkable differences between Europe and the Maghreb with this split being dated back to the Messinia Salinity Crises more than 5 My ago [31]. Having in mind the differentiation pattern in all known marker systems, we believe that these two mtDNA lineages in the entire species complex might have originated at that time horizon, but were distributed to different geographical regions only much later by lineage sorting, maybe hereby exemplifying one case of the often observed difference between mtDNA on the one hand and nuclear DNA sequences, morphological characteristics and allozyme pattern on the other [32].

Our allozyme data further show strong differentiation within *M. galathea *into two major groups with respective subgroups: (i) Sicily - Tunisia with (i-a) Sicily and (i-b) Tunisia as well as (ii) all other *M. galathea *with (ii-a) Morocco, (ii-b) Italy with parts of southern France, (ii-c) western Balkan including the western Carpathian Basin, (ii-d) eastern Balkans with Romania and Central Europe, and (ii-e) the south-western Alps.

### Atlantic-Mediterranean origin of the *M. galathea/lachesis *species complex

**Figure 4:**
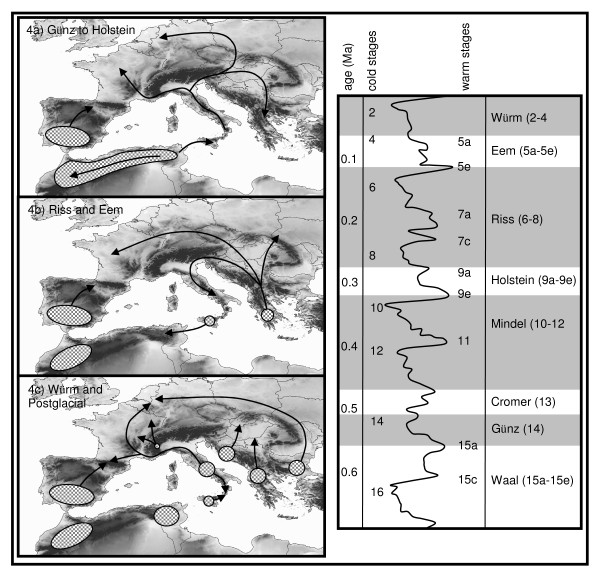
**Biogeographic scenario of *Melanargia galathea *and *M. lachesis***. Range expansions and retractions of *M. galathea *and *M. lachesis *(on the Iberian Peninsula) during the past ice-ages (a-c) and fluctuations of marine isotope stages (d) (redrawn after Gibbard & van Kolfschoten [33]). Refugia are marked by grey areas, expansions/retractions by arrows. 4a) Günz and Mindel refugia in Iberia (*M. lachesis*) and the Maghreb (*M. galathea*); expansion from the eastern Maghreb to Sicily during the Mindel/Holstein transition; Holstein expansion over Europe, but retraction in the Maghreb to the west. 4b) Riss refugia in Iberia (*M. lachesis*) and the western Maghreb, Sicily and southern Balkans (*M. galathea*; Riss/Eem transition expansion from Sicily to the eastern Maghreb; Eem expansion of the southern Balkan group over major parts of Europe including peninsular Italy. 4c) Würm refugia in Iberia (*M. lachesis*) as well as western and eastern Maghreb, Sicily, peninsular Italy, southwestern Alps and Balkan area (*M. galathea*); postglacial expansion from Iberia (*M. lachesis*), peninsular Italy and the Balkan area (*M. galathea*).

The recent geographic restriction of *M. lachesis *to Iberia and the highest genetic diversity of *M. galathea *in Morocco support the idea of a centre of origin of the entire species complex in this area. This assumption is further supported by other *Melanargia *species mostly endemic to the Atlantic-Mediterranean region (*M. occitanica, M. ines*) and other endemics to further Mediterranean refugia (*M. arge*: peninsular Italy; *M. pherusa*: Sicily, *M. larissa*: Pontic-Mediterranean region and Iran). The onset of the differentiation between these sister species should be due to vicariance events most likely correlated with the onset of an ice age. If giving one glacial-interglacial cycle for the lowest level of differentiation (i.e. the subgroups within the two major *M. galathea *lineages), the most likely time horizon of this vicariance event is the onset of the Günz glaciation some 560,000 years BP [33] (Figure 4a). Since then, *M. lachesis *most likely has never expanded out of Iberia whereas *M. galathea *colonised most of Europe from its Maghreb expansion centre. Similar splits between Iberia and the Maghreb are commonly observed in many species groups [e.g. [13,34-36]].

### From the Maghreb to Europe

The deepest split in the *M. galathea *populations is between the Sicily - Tunisia group and all the other populations. As this split is about twice the genetic differentiation among their subgroups and less than half of the distance against *M. lachesis*, the onset of the Riss glaciation (about 310 ky BP) [33] might be the trigger for vicariance and thus the beginning of this differentiation. As (i) Iberia was continuously blocked for the expansion of *M. galathea *to Europe by *M. lachesis *[cf. 27] and (ii) all European *M. galathea *populations except Sicily are more similar to populations from Morocco than from Tunisia, a scenario with this split taking place in the Maghreb is little likely. This assumption is further supported by SDMs for ice age conditions predicting mostly continuous distributions over North Africa (Figure 3b) thus allowing vicariance in this region only during the relatively short interglacial stages. For these reasons, *M. galathea *must have reached Europe before the Riss glaciation.

As the region of the eastern Sahara in Egypt apparently always have been too dry for an expansion of *M. galathea*, this first expansion of *M. galathea *to Europe must have been from Tunisia to Sicily (Figure 4a), a sea strait known for biogeographical connections for many taxa [e.g. [15]; and references therein]. As the Strait of Sicily was considerably narrower during glacial periods due to eustatic sea level lowering, the transition from Mindel glaciation to Holstein interglacial with still low sea level but already higher temperatures might have been a suitable time period for this dispersal. After arrival to Sicily, the Holstein interglacial might have given suitable condition for the expansion of *M. galathea *over most parts of Europe, including the Balkans but excluding Iberia as this peninsula was already populated by *M. lachesis *(Figure 4a).

With the climatic cooling of the Riss ice age, which was considerably longer than the following Würm glaciation and had longer durations of minimum temperatures [33,37], *M. galathea *most probably was nearly extinct in Europe only surviving in the southernmost possible retreats in Sicily and the southern Balkans (Peleponnesos), but also in the Maghreb; *M. lachesis *could survive in southern Iberia (Figure 4b). This vicariance might be the origin of the two major European lineages of *M. galathea *with the eastern one by chance evolving similarly in allele frequencies as the Morocco lineage, with this similarity therefore not representing recent biogeographical connection between them. Riss vicariance events most likely have also been responsible for other differentiation processes as e.g. in the *Polyommatus coridon */*hispana *complex [e.g. [38]].

### ...and back to the Maghreb

As the time for differentiation between the four *M. galathea *lineages from continental Europe is assumed to be the result of one glacial cycle (see above) and as the differentiation between populations from Sicily and Tunisia are in the same order of magnitude, we assume that the onset of this differentiation is in the same time frame. As the genetic diversity is significantly higher in Sicily than in Tunisia and the warm and dry interglacial climatic conditions in Tunisia generally unsuitable for the survival of *M. galathea*, we assume that a colonisation most likely has taken place from Sicily to Tunisia. While the sea level was still considerably lowered at the transition from Riss to Eem thus facilitating dispersal between these two areas, this time period might be the most likely for this expansion event. During the following Eem interglacial, the Balkan refuge of *M. galathea *most probably could colonise most parts of Europe apart from Iberia and Sicily, which were occupied by other genetic lineages of this species complex (Figure 4b).

### The existence of extra-Mediterranean refugia for thermophilic taxa

During the Würm ice age, which was not more severe than the two previous glaciations but with a shorter maximum [33], the Marbled White butterflies were not that much pushed to the South than in the previous cases. This is well matching the remarkable differentiation of the species in Europe allowing to distinguish five lineages (see above), which most likely are the result of survival of the Würm ice age in a larger number of different refugia.

This pattern implies at least two different refugia at the Balkan Peninsula at the western and the eastern flank; more in detail analyses also support a third Balkan centre in the peninsula's southern parts [29] (Figure 4c). This pattern of multiple refugia in the Balkans was already erected by Reinig [39] postulating different centres of survival in the western, southern and eastern Balkans and was later supported by genetic analyses showing genetic divergences between these areas for a variety of different animal species [e.g. [18,40-42]].

Furthermore, different Würm refugia have to be postulated for Sicily and peninsular Italy, a pattern also repeated by other genetic analyses [e.g. [17,43]]. Furthermore, other genetic studies show a remarkable genetic differentiation in the southernmost parts of peninsular Italy [e.g. [34,44,45]].

The last remaining lineage of *M. galathea *in the south-western Alps most likely is not representing a Mediterranean refuge of this species, but an extra-Mediterranean refuge area at the southern slopes of the glaciated Alps (Figure 4c). As already shown by Steward and Lister [46], glacial survival of temperate species in Europe was not only possible in the classical Mediterranean refugia sensu de Lattin [47], but also in small climatically buffered pockets in more northern regions [8,48,49]. Recent works especially highlight the southern and south-eastern parts of the Alps of particular importance for additional Würm ice age refugia for temperate species [e.g. [42,50,51]], and also for species formerly thought to be of exclusive Mediterranean origin [e.g. [52,53]]. This apparently was also the case for the Marbled White.

### Postglacial expansion

During the Postglacial, several lineages of *M. galathea *were mostly blocked in their expansion by other lineages representing the respective leading edges [cf. 54]. In the case of *M. galathea *in Morocco, their northwards expansion was blocked by *M. lachesis *distributed in Iberia. The lineage surviving in the eastern Balkans apparently had the most important impact in the recolonisation of more northern parts of Europe as its dispersal was not hampered by any major mountain obstacle [cf. 9] so that this lineage could expand throughout Central Europe to the western parts of Germany (Figure 4c). However, the samples of north-eastern France and southern Germany show an intermediate genetic structure between this lineage and the south-western Alps lineage, making hybrid origin of these populations rather likely and thus expansion of the southern Alps lineage over the chains of the Alps.

Also the Italian lineage could expand beyond its refugium to southern France. Therefore the entire region of northern France and southern Germany might be a zone of mixing between these three lineages. Hybrid zones between different taxa are frequently observed in this region [e.g. [9,55]]. Furthermore, the southernmost population in Calabria (southern Italy) has an intermediate genetic texture between the Italian and the Sicily group thus speaking for a postglacial contact and intermixing between these two groups in this region.

## Conclusion

The hierarchical structure of our allozyme data set on *M. galathea *and *M. lachesis *is consistent with the chronology of the last four glacial-interglacial cycles. Based on this consistency, we derive the following scenario, which in our opinion is the most likely one: (i) The beginning of the Günz ice age might have affected the vicariance between the two species. (ii) *M. galathea *might have crossed from Tunisia to Sicily at the transition from Mindel ice age to Holstein interglacial and (iii) subsequently spread all over Europe, but retreated in the Maghreb to the higher elevations of the Atlas mountains. (iv) The members of this species complex survived the coldest periods of the Riss glaciation only in southern Iberia, Morocco, Sicily and the southern Balkans (Peleponnesos). (v) At the transition from Riss ice age to Eem interglacial, Tunisia was recolonised from Sicily. (vi) The southern Balkan group might have colonised major parts of Europe during the Eem interglacial including Italy and Central Europe. (vii) Populations of this group survived the Würm ice age in Italy, the southern margin of the Alps, the western and eastern flank of the Balkan peninsula; members of other lineages survived in Sicily, Tunisia, Morocco and Iberia. (viii) During the Postglacial, only the eastern Balkan and the Italian lineage showed major northwards range expansion. (ix) Hybridisation between lineages most probably occurred in western Central Europe and southern Calabria.

## Methods

### Allozyme electrophoresis

**Figure 5:**
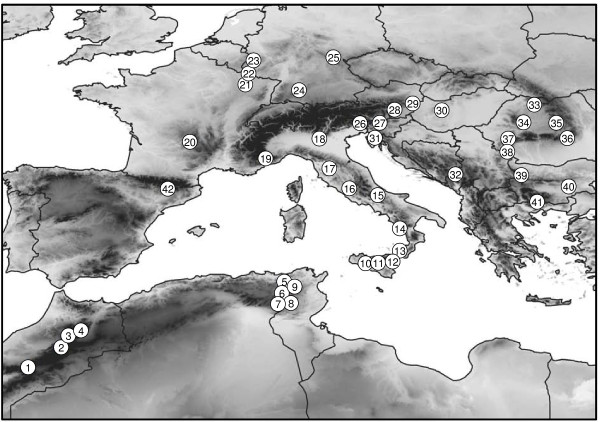
**Sampling design of *Melanargia galathea *and M. *lachesis***. Geographical location of the sampled populations of *M. galathea *and *M. lachesis *(sample 42). Given numbers coincide with Table 1.

We scored banding patterns of allozyme polymorphisms analysed for 1,463 *M. galathea *specimens from 41 populations sampled across major parts of the Maghreb and Europe and one populations of *M. lachesis *(40 individuals) from the Spanish Pyrenees (see Figure 5 and Table 1). In total we analysed 15 enzyme systems: 6PGDH, ACON, FUM, G6PDH, GAPDH, AAT2, GPDH, PGI, HBDH, IDH1, IDH2, MDH1, MDH2, ME, and PEP. The data of 26 *M. galathea *populations and of *M. lachesis *were taken from Habel et al. [27,28] and Schmitt et al. [29]. Specimens were netted in the field from mid-May to the beginning of August between 2004 and 2010, frozen alive in liquid nitrogen or in a freezer and stored under these conditions until analysis. Standard procedures of allozyme electrophoresis were performed as described in Habel et al. [27].

### Statistics

Alleles were labelled according to their relative mobility, starting with "1" for the slowest. All laboratory results were stored on cellulose acetate plates. These banding patterns were (re)analysed by one person (JCH). Allele frequencies, Nei's standard genetic distances [56] and parameters of genetic diversity (i.e. mean number of alleles per locus, *A*, expected heterozygosity, *H*_e_, and observed heterozygosity, *H*_o_, total percentage of polymorphic loci, *P*_tot_, and percentage of polymorphic loci with the most common allele not exceeding 95%, *P*_95_) were computed with G-Stat [57]. As sample sizes do not differ significantly, the calculation of allelic richness correcting for population sizes was not necessary. For detecting differences of means of genetic diversities among genetic lineages and sublineages, we calculated U-tests using STATISTICA. Conventional F statistics, AMOVAs, hierarchical genetic variance analysis, tests of Hardy-Weinberg equilibrium and linkage disequilibrium were calculated with ARLEQUIN 3.1 [58]. Phenograms using the neighbour joining algorithm [59] were constructed with PHYLIP [60], including bootstrap-values (calculated based on 1,000 iterations). To define individual based genetic clusters we performed STRUCTURE analyses [61]. As burn-in and simulation lengths we used 100,000 and 300,000 iterations per run based on the admixture model with correlated gene frequencies comparing different groupings (from K = 2 to K = 10).

### Species Distribution Modelling

Over the last few decades, Geographic Information System (GIS) based Species Distribution Models (SDMs) have become vital tools used to predict the potential distribution of species under current conditions and climate change scenarios [62-64]. In combination with palaeoclimatological data, SDMs have been suggested as a mean of inferring species' past distributions [65,66], especially when combined with phylogeographic techniques [67].

We compiled a set of 3,483 species records of *M. galathea *from online data bases (Global Biodiversity Information Facility - GBIF; http://www.gbif.org) and our own field surveys. The accuracy of all records was checked in DIVA-GIS 5.4 [68] and only those which could be unambiguously assigned to a single grid cell with a resolution of 2.5 arc min (ca. 4 km in the study area) were used for further processing. Since unequal spatial clumping of species records may cause problems when computing SDMs, the species records were filtered in geographic space, leaving only 1 record per 10 arc min. The final data set comprised 535 records (Figure 3a) scattered all over the known range of the species in Europe and North Africa.

We obtained information on current and past climate as described by the Community Climate System Model (CCSM; http://www.ccsm.ucar.edu) with a spatial resolution of 2.5 arc min from the Worldclim data base ([69]; http://www.worldclim.org). Original palaeoclimatological data were previously processed as described by Peterson and Nyári (2007) [70]. A total of 19 BIOCLIM variables were previously suggested as suitable for SDM computation [71,72]. However, inclusion of too many inter-correlated variables or biologically irrelevant predictors may hamper the transferability of SDMs through space and time [73-76]. Therefore, we first computed a pair-wise correlation matrix based on Pearson's correlation coefficients among all 19 predictor variables and excluded those with *R^2 ^*> 0.75. Subsequently, we chose a final set of eleven predictors describing biologically relevant climate conditions for the long-term persistence of *M. galathea *populations (i.e. annual mean temperature, maximum temperature of warmest month, minimum temperature of coldest month, temperature annual range, mean temperature of wettest quarter, mean temperature of driest quarter, annual precipitation, precipitation of wettest quarter, precipitation of driest quarter, precipitation of warmest quarter, precipitation of coldest quarter).

For SDM computation, Maxent 3.3.2 was applied [77,78] using the default program settings. Random background records were automatically sampled by Maxent within the study area. Species records were split 100 times into 70% used for model training and 30% for model evaluation via the area under the receiver operating characteristic curve (AUC; [79]). Subsequently, the average predictions for current and past conditions of the logistic output of the 100 models were computed and transformed into presence/absence maps applying the average minimum training presence and average 10% training omission as thresholds.

## Authors' contributions

JCH sampled and analysed *M. galathea*-individuals, performed statistics, data-interpretation, and has written major parts of the manuscript. TS sampled individuals, and supported the work by data-interpretation and writing. DR performed species distribution models, and LL strengthened the significance of the manuscript by data-interpretation and by upgrading the linguistic style. All authors red and approved the final version of the manuscript.
